# Agro-morphological and biochemical responses of quinoa (*Chenopodium quinoa* Willd. var: ICBA-Q5) to organic amendments under various salinity conditions

**DOI:** 10.3389/fpls.2023.1143170

**Published:** 2023-05-08

**Authors:** Ayoub El Mouttaqi, Talal Sabraoui, Mohamed Belcaid, Mohamed Ibourki, Ihssane Mnaouer, Karima Lazaar, Faissal Sehbaoui, Reda Ait Elhaj, Manal Khaldi, Sifeddine Rafik, Jamaâ Zim, Abdelaziz Nilahyane, Cherki Ghoulam, Krishna Prasad Devkota, Lamfeddal Kouisni, Abdelaziz Hirich

**Affiliations:** ^1^ Agriculure in Marginal Environment Program, African Sustainable Agriculture Research Institute (ASARI), Mohammed VI Polytechnic University (UM6P), Laayoune, Morocco; ^2^ Agri-Edge, Mohammed VI Polytechnic University (UM6P), Benguerir, Morocco; ^3^ Department of Food Science and Agricultural Chemistry, McGill University, Sainte-Anne-de-Bellevue, QC, Canada; ^4^ Department of Plant Protection, Agronomic and Veterinary Institute Hassan II, Agadir, Morocco; ^5^ AgroBioSciences Program, Mohammed VI Polytechnic University, Ben Guerir, Morocco; ^6^ Agrobiotechnology & Bioengineering Centre, Cadi Ayyad University, FST, Marrakech, Morocco; ^7^ Soil, Water, and Agronomy (SWA) Program, International Center for Agricultural Research in the Dry Areas (ICARDA), Rabat, Morocco

**Keywords:** quinoa, osmotic stress, organic amendment, growth, seed yield, saponin

## Abstract

In the Sahara Desert, due to drought and salinity and poor soil fertility, very limited crop choice is available for the farmers to grow crops. Quinoa (*Chenopodium quinoa* Willd.) has shown promising under such conditions in the South of Morocco, a true representative site of Sahara Desert. Soil organic amendments have the potential to minimize negative effects of soil salinity and improve crop production. Thus, this study aimed to elucidate the impact of nine organic amendments on quinoa (var. ICBA-Q5) growth, productivity, and biochemical parameters under saline irrigation water application (4, 12, and 20 dS·m^-1^). Results of the experiment indicate a significant effect of organic amendments on major agro-morphological and productivity parameters. Biomass and seed yield tends to decrease with the rise of salinity level, and organic amendments have improved productivity compared to the non-treated control. However, salinity stress alleviation was assessed by determining pigments concentration, proline content, phenolic compounds, and antioxidant activity. Therefore, the action of organic amendments varies from one level of salinity to another. Furthermore, a remarkably significant decrease in total saponin content was reached due to the application of amendments even at high saline conditions (20 dS·m^-1^). The results demonstrate the possibility of enhancing the productivity of quinoa as an alternative food crop under salinity conditions by using organic amendments and improving the quality of grains (saponin reduction) during the pre-industrialization process.

## 1 Introduction

Crop production is threatened and limited by various challenges including salinity. FAO (2021) estimated that salinity affects more than 424 million hectares of topsoil (0-30 cm) and 833 million hectares of subsoil (30-100 cm). In Africa, 80 million hectares of lands are saline, sodic, or saline-sodic, of which the Sahel, in West Africa, is the most affected (Shahid et al., 2018). Salinization occurs under all climatic conditions and can result from both natural (primary salinization) and human-induced actions (secondary salinization). In agricultural area, secondary salinization is the most dominant form of salinity and is caused by inadequate irrigation, misuse of fertilizers, over-pumping, improper drainage, seawater intrusion, etc. In general, salinization occurs in arid and semi-arid regions where precipitation is very low, and evaporation is high which can result in salt accumulation in the rootzone and lack of leaching (Stavi et al., 2021). Salinity affects plants and reduces their growth and productivity by limiting water and nutrient uptake (osmotic stress), accumulating toxic ions such as sodium, reducing transpiration and photosynthetic activity, damaging cell membranes and causing plant senescence (Machado and Serralheiro, 2017). Salt-tolerant plants have the ability to deal with salinity by deploying several mechanisms including osmotic adjustment, sodium compartmentalization, salt exclusion, etc (Volkov and Beilby, 2017).

Quinoa (*Chenopodium quinoa* Willd.) is a good example of salt-tolerant crops that recently attracted a lot of attention worldwide thanks to its wide adaptation to various climatic and soil conditions, rusticity and resilience, and its high nutritional value. Quinoa originates from the Andean countries, where it was domesticated over 7000 years ago by the local people. Quinoa was neglected for many centuries following the Spanish conquest and was geographically limited to the Andes, where it was a basic staple food for the indigenous people (Bazile et al., 2016). The potential of quinoa was rediscovered during the second half of the 20^th^ century and since then, quinoa was tested and introduced to over 120 countries around the world (Alandia et al., 2020). Peru and Bolivia are both leading exporters of quinoa grains worldwide, contributing 78% of the total export (exporting 44,353 and 29,416 tons, respectively, out of a total of 93,809 tons/year) (Alandia et al., 2020; Schmidt et al., 2021).

Quinoa was first introduced in Morocco in 2000 and since then its value chain significantly improved. In the beginning of its introduction, research works mainly focused on quinoa agronomic adaptation (Jellen et al., 2006), diversity screening (Mhada et al., 2014), irrigation (Hirich et al., 2014a; Hirich et al., 2014b; Fghire et al., 2015), organic amendment (Hirich et al., 2014b), salinity (Brakez et al., 2013; Hirich et al., 2014c), and sowing date (Hirich et al., 2014d). Recent research is oriented towards production transformation (Mhada et al., 2020; Hirich et al., 2021; Rafik et al., 2021b, 2021c), marketing (Rafik et al., 2021a), value chain analysis (Hirich et al., 2021; Rafik et al., 2021a), etc.

In order to enhance tolerance level to salinity in quinoa plants and improve their yield, it is necessary to increase the nutrient and water availability in the soil and their absorption through the application of organic amendments or fertilizers. Organic amendments were shown to be highly beneficial for crop production, especially under stress conditions (Leogrande and Vitti, 2019). In fact, by adding organic matter to the soil, the nutrient availability for the plant and water holding capacity increases, and the soil’s biological activity gets improved. Another role of organic amendments is they improve soil structure and properties resulting in accelerating salt leaching in the rootzone (Ding et al., 2020; Wichern et al., 2020).

To mitigate the drastic negative effects of abiotic stress such as salinity, halophytes have developed an array of salt-tolerance mechanisms by controlling the uptake and canalization of Na^+^, K^+^ and Cl^-^ and the biosynthesis of osmolytes (Flowers and Colmer, 2008). Quinoa is one of the famous halophytes that contains a good balance of starch, protein, lipids and fiber, minerals, and also contains a good amount of non-nutritional compounds such as saponin, polyphenols, flavonoids, carotenoids, that control various biological activities (Miranda et al., 2012; Gómez-Caravaca et al., 2014).

Saponins are bitter, soapy substances that protect quinoa plant from fungal disease and insect attacks. They also contain toxins that can cause irritation and other health issues to some people (Liao et al., 2017). While the level of toxicity is low, some people may be sensitive to this compound. Saponins are also considered secondary metabolites with numerous health promoting compounds (Puente-Garza et al., 2017; Lim et al., 2020; Yi, 2021) commonly used in the agro-food industry as food additives, and emulsifiers in cosmetic products. While in some cases, saponins could be harmful for human consumption due to their weak absorption, and toxic effect (Yi, 2021).

Nevertheless, little information is available so far about the influence of organic amendments on saponins content. Therefore, the aim of this study is to investigate the influence of organic amendments on plant growth, seed yield, and grain quality parameters such as saponins, phenolic contents, and antioxidant activity in quinoa under different salinity levels.

## 2 Materials and methods

### 2.1 Experimental site

The study was conducted at the experimental farm of National Institute of Agricultural Research in Foum El Oued area, Laayoune, south of Morocco (X = -13.341°; X = 27.186°; Z = 13 m). The soil in the experimental site was sandy loam, characterized by strong salinity and low content of organic matter and nutrients (**Table 1**).

**Table 1:** Initial soil physical and chemical properties in the experimental site.

Sand (%)	Silt (%)	Clay (%)	pH	ECe (dS·m^-1^)	OM (%)	N (%)	P_2_O_5_ (ppm)	K_2_O (ppm)	MgO (ppm)	CaO (ppm)	Na_2_O (ppm)	Cl (ppm)	Zn (ppm)	Fe (ppm)
61.8	19.6	18.6	8.48	9.55	0.47	0.03	44.12	330	920	9730	2000	2120	0.8	1.23

### 2.2 Experimental design and treatments

**Figure 1:**
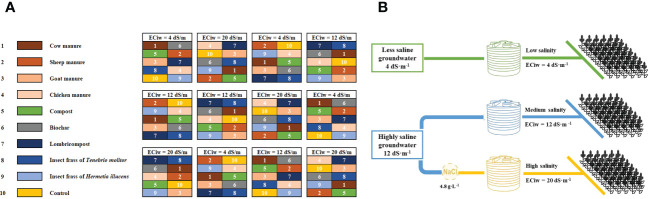
Experimental design showing allocation of main and sub-plot treatments **(A)**, Layout of the irrigation water distribution system in the experimental plots **(B)**.

The experiment was conducted using a split-plot design with four replications. The salinity treatment was applied in the main plot, and the organic amendments were applied in the subplots. The area of each plot was 15 m^2^, each consisting of ten rows (**Figure 1A**). The crop was sown manually on April 13th, 2021, using the ICBA-Q5 quinoa variety. Irrigation was applied using a drip irrigation system (row-to-row spacing: 50 cm, distance between drippers: 20 cm, and flow rate: 2L/hr). The crop was irrigated twice a week and received a total irrigation amount of 240 mm during its growing cycle.

Irrigation water salinity treatments were applied by filling water tank with two sources of groundwater for low (4 dS·m^-1^) and medium salinity level (12 dS·m^-1^), which corresponds to the lowest and the medium groundwater salinity level in the region, respectively. While 20 dS·m^-1^ was made by adding sodium chloride to the medium salinity treatment (**Figure 1B**).

Three irrigation water salinity levels, i.e., 4, 12 and 20 dS·m^-1^ were applied in main-plots. Characteristics of the irrigation water used in this experiment are presented in **Table 2**.

**Table 2:** Chemical properties of irrigation water applied.

Water source	ECiw (dS·m^-1^)	pH	Cations (ppm)				Anions (ppm)				
			K	Na	Ca	Mg		Cl	SO_4_	NO_3_	HCO_3_
Low saline groundwater	4.04	7.45	34.52	559.81	225.45	78.73		996.85	538.42	214.52	214.79
High saline groundwater	11.98	7.35	134.5	2622.47	569.14	321.01		4415.3	2504.76	62.62	236.76

Nine different organic amendments in addition to a non-treated control were tested in sub-plots. Their characteristics and applied doses are presented in **Table 3**. The application rate of organic amendments was based on the local farmers’ practices and the cost of the products in the region.

**Table 3:** Applied organic amendment with their doses and chemical properties.

Element	Cow manure	Goat manure	Sheep manure	Chicken manure	Compost	Insect frass of *Hermetia illucens*	Insect frass of *Tenebrio molitor*	Lombri-compost	Biochar
Applied dose (t·ha^-1^)	30	30	30	5	5	5	5	5	5
EC 1:5 (dS·m^-1^)	24.08	19.22	19.92	15.81	8.21	15.19	6.47	2.07	0.66
pH	7.98	7.95	8.24	7.12	7.57	7.05	5.97	8.6	7.9
Nitrogen (%)	1.51	1.89	1.51	3.68	2.17	1.18	1.16	0.23	0.52
Carbon (%)	29.38	32.91	25.71	16.9	27.59	4.74	4.45	4.65	11.74
C/N ratio	19.46	17.41	17.03	4.59	12.71	4.02	3.84	20.21	22.58
Phosphorus (%)	0.41	0.58	0.37	0.97	0.65	1.6	1.08	0.26	0.07
Potassium (%)	1.85	2.86	3.67	2.45	3.21	2.37	1.99	0.52	0.49
Calcium (%)	5.45	3.89	2.88	1.56	3.97	0.93	0.58	7.47	3.18
Magnesium (%)	1.12	1.07	0.97	0.76	1.91	0.47	0.62	0.63	0.18
Copper (ppm)	120.58	158.66	29.64	887	49.99	47.85	23.17	473.12	16.76
Manganese (ppm)	143.08	161.48	100.11	382.67	544.30	154.82	297.94	180.29	796.08
Iron (ppm)	2666.31	4054.86	2508.04	953.32	14117.10	899.54	252.92	13860.71	3425.04
Zinc (ppm)	268.05	374.52	77.32	692.67	175.47	222.08	172.11	329.18	117.94
Boron (ppm)	97.99	98.05	132.94	50.48	136.53	25.74	17.95	131.83	61.47

### 2.3 Climatic condition

**Figure 2:**
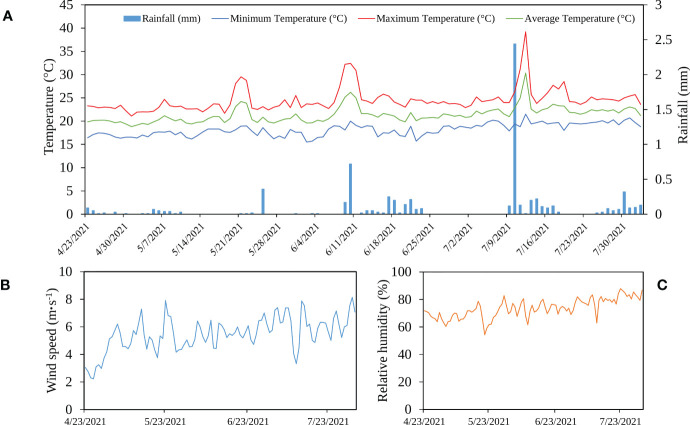
Variation of rainfall and temperature **(A)**, wind speed **(B)**, and relative humidity **(C)** during the growing period in Laayoune, Morocco.

**Figure 2** shows the variation of different climate parameters measured at the local weather station in the experimental site. The crop received a total amount of rainfall equal to 7 mm. Temperature and relative humidity were almost stable during the growing season, with 2 picks of high temperatures exceeding 30°C. The wind speed increased during the growing period (from April to July) and had an average speed of 5.5 m·s^-1^.

### 2.4 Measurement parameter

#### 2.4.1 Morphological traits

Four individual representative plants (sampled from the average height of randomly measured 10 plants) per plot were harvested (with root) at the beginning of the seed-filling stage, when leaves were still in the plants. All plant organs, including roots, stems, leaves, and panicles were separated and weighed (fresh weight). Root volume, length, and width of different organs were recorded, and then all plant materials were oven dried for 48 hours at 60°C to obtain a constant dry weight. Phenological observation such as days to appearance of 1^st^ flowers; and the number of flowering plants were counted every 2 days interval until reached the 50% of the whole subplot were measured.

#### 2.4.2 Crop productivity

The harvest was manually carried out for each row on July 29th, 2021. Fresh biomass was first determined, then the harvest product was oven dried until a constant weight of dry biomass was reached. Afterward, the biomass was threshed, and grains were collected, cleaned, and weighed to determine the seed yield. The harvest index (HI) was calculated as the ratio of seed yield to total dry biomass. Furthermore, crop water productivity (CWP) was calculated by dividing the seed yield by the total irrigation amount applied (also with rainfall).

#### 2.4.3 Soil salinity and humidity

Soil electrical conductivity (ECe) and humidity were measured using Decagon TEROS 10 and 12 at the rootzone depth of 15 cm. Data were recorded every 15 minutes and stored in the Decagon ZL6 data logger. Soil salinity and humidity sensors were placed for sheep manure application’s plot under three irrigation water salinity treatments. While, soil moisture sensors were installed for biochar, compost, and sheep manure plots under low salinity treatment (4 dS·m^-1^) to compare the soil water retention for this three amendments.

#### 2.4.4 Chemical analysis

Samples of soil, irrigation water, and organic amendments were analyzed in the AITTC-UM6P (Agricultural Innovation and Technology Transfer Center-Mohammed VI Polytechnic University) central laboratory following the Kjeldahl method for nitrogen, ICP-OES (Inductively Coupled Plasma-Optical Emission Spectrometry) for other elements such as phosphorus, calcium, magnesium, potassium, copper, manganese, iron, zinc, and boron. While the bicarbonates (HCO_3_) concentrations in the irrigation water were measured using the method ISO 9963-1 (ISO, 1994).

#### 2.4.5 Biochemical content

##### 2.4.5.1 Chlorophyll a and chlorophyll b content

Chlorophyll a (Chl a), Chlorophyll b (Chl b), and total chlorophyll were analyzed using the methodology as described by Pérez-Patricio et al. (2018). 0.5 g of fresh quinoa leaves were macerated under low luminosity conditions. The macerate was mixed with 4 mL of 99% acetone and 2 mL of ethanol (2:1 v/v), then stirred for 1 min. After 30 minutes in a dark freezer, the solution was centrifuged at 2000 rpm for 10 minutes, then 5 mL of acetone/ethanol (2:1 v/v) was added to the mixture and stirred for 1 min. The spectrophotometer was used to measure photosynthetic pigments at 663 nm and 645 nm. The solution of acetone/ethanol (2:1 v/v) was used as a blank solution. The following equations are used to calculate the concentration of chlorophyll a and b pigments:


$$Chl\;a\;\left(mg\cdot g^{-1}\right)=\;\left(12.72\;*\;A663\right)-\;\left(2.59\;*\;A645\right)$$

$$Chl\;b\;\left(mg\cdot g^{-1}\right)=\;\left(22.88\;*\;A645\right)-\;\left(4.67\;*\;A663\right)$$

$$Total\;chlorophyll\;\left(mg\cdot g^{-1}\right)=Chl\;a+Chl\;b$$

A645 and A663 represent the absorbance values at 645 nm and 663 nm, respectively.

##### 2.4.5.2 Leaf mineral content

The determination of mineral content of quinoa leaves was performed according to the protocol as described by Pequerul et al. (1993). The dried leaf samples were finely ground and sieved through a 2 mm pore sieve. Samples were further prepared by adding 8 ml of nitric acid (HNO3) to the grounded material (0.5 g), it was placed in a digestion tube and left overnight. The mixture was then heated at 90°C for 60 minutes and 3-4 ml of 30% hydrogen peroxide (H2O2) was added. The digestion process was stopped once the solution became colorless, and after cooling, the digest was filtered and diluted with hydrochloric acid (HCl). The elemental determination of sodium and chloride in extracted samples was carried out by using a flame photometer (FP902, PG Instruments).

##### 2.4.5.3 Leaf proline content

The proline content of quinoa leaf was determined as described by Bates et al. (1973) with minor modifications. Briefly, 0.5 g of grounded quinoa leaf was weighed and homogenized with 3% 10 mL of sulfosalicylic acid. After that, 2 mL of the mixture was added to 2 mL of glacial acetic acid, and 2 mL of 2% ninhydrin solution were added. The mixture is incubated in a water bath at 95°C, after cooling in an ice bath, proline was extracted with 4 mL of toluene, the mixture is discarded, and the top layer was extracted, and its absorbance was measured at 520 nm. Proline concentration was calculated using a calibration curve of L-proline (0-40 µg/mL) and expressed as µmole Proline/g of fresh weight.

##### 2.4.5.4 Extraction of bioactive components

Saponins extraction was carried out using the method of (Navarro del Hierro et al., 2018) with slight modifications. Briefly, 5 g of each quinoa seed’s powder was placed in a filter paper cartridge and defatted using the Soxhlet apparatus with Hexane (1:10 w/v) as solvent. Ultrasound-assisted extraction of saponins was performed with methanol (1:10 w/v). Extraction was carried out using an ultrasonic probe at 60% amplitude for 15 min (3 cycles of 5 min). The mixture was filtered through Whatman paper N°1. The mixture was centrifuged, and the supernatant was dried under vacuum and then reconstituted in 5 mL of methanol.

##### 2.4.5.5 Total Saponin Content (TSC)

The total saponins content was determined using the modified method of Lim et al. (2020). 0.25 ml of saponin extract was added to 1 mL of reagent mixture (glacial acetic acid/sulfuric acid 1:1 v/v) and vortexed. After that, the mixture was incubated at 60°C in a water bath for 30 min and then placed in an ice bath to cool. The absorbance of the sample was read at 527 nm using a UV-visible spectrophotometer (VWR International, USA). Oleanolic acid was used to prepare the calibration curve (0 – 1000 μg/mL). Total saponins content was expressed as g of oleanolic acid equivalent/100 g DW.

##### 2.4.5.6 Total Phenolic Content (TPC)

Total phenolic content was determined as reported by Gómez-Caravaca et al. (2011). Aliquots of 200 mL of each sample were mixed with 1 mL of 10% Folin reagent and left to stand for 6 min at room temperature in the dark. Then, 800 mL of Na2CO3 7.5% (w/v) was added to the mixture and placed in the dark to react for 30 min before measuring the optical densities at 750 nm. A control was prepared also and gallic acid was used as a reference standard to prepare the calibration curve. The total phenolic content was expressed as mg gallic acid equivalent (GAE)/100 g of DW.

##### 2.4.5.7 Antioxidant activity (AOX)

Determination of the scavenging activity of quinoa extract was conducted using the 2.2-diphenyl-2-picryl-hydrazyl (DPPH) assay according to the procedure previously described by Fischer et al. (2013). Briefly, 4.9 mL of a 0.1 mM methanolic solution of DPPH (violet color) was mixed with 100 μL of different concentrations of quinoa extract. The tubes were allowed to react in the dark for 30 min at room temperature. The loss of violet color of the mixture was measured at 517 nm. A positive control containing methanol instead of the sample was prepared and Trolox (0-12 µmole) was used to prepare the standard curve. Results are expressed as µmol of Trolox per gram of extract (µmol TE/g E).

The method described by Yang et al. (2022) was used to measure the activity of scavenging ABTS radical cation (ABTS+). Initially, a stock solution of ABTS was created by mixing 7.4 mM of an aqueous solution of ABTS with 2.6 mM of an aqueous solution of potassium persulfate and allowing it to sit in the dark at room temperature for 12 hours. The ABTS+ cation solution was then diluted with methanol until its absorbance value was 0.7 ± 0.02 at 734 nm. Next, 100 μL of phenolic extracts were added to 2 mL of the ABTS+ cation solution and incubated in the dark for 10 minutes at room temperature. Finally, the absorbance of the mixture was measured at 734 nm. Results are expressed as µmol of Trolox equivalents per gram of extract (µmol TE/g E).

The modified method of Lim et al. (2020) was used to determine the reducing power of quinoa extract. In brief, each sample’s extract (0.5 ml) was mixed with 1.25 ml of 0.2 M phosphate buffer (pH 6.6) and 1.25 ml of 30 mM potassium hexacyanoferrate (III) and incubated at 68°C for 20 min. Then, 1.25 ml of 0.6 M trichloroacetic acid was added, and the mixture was centrifuged at 4032xg for 10 min. The supernatant was collected, and 1 ml of distilled water and 0.2 ml of iron (III) chloride were added to 1 ml of the supernatant. This mixture was kept at room temperature for 15 min, and the absorbance of the final test solution was measured at 700 nm using a spectrophotometer. Results are expressed as µmol of Trolox equivalents per gram of Dry weight (µmol TE/g DW).

### 2.5 Statistical Analysis

Statistical analysis was performed using the statistical programming language R 4.1.3. The additive model of the analysis of variance (ANOVA) was used to assess the effects of salinity and organic amendments on the monitored parameters. Then, Tukey’s multiple comparison tests was conducted to appraise the significant differences between the mean values at a 5% level of significance. Pearson’s correlation matrix was performed to investigate the strength of the linear relationship between variables and visualized using the “corrplot” package. Principal component analysis (PCA) was utilized to examine the correlation among the traits and evaluate the impact of the factors on the identified correlation patterns. To perform the PCA, the “factominer” and “factoextra” packages were used, and the factors were projected as supplementary qualitative variables.

## 3 Results

### 3.1 Agro-morphological traits

Variations of different monitored morphological traits of quinoa as affected by salinity level and organic amendments are presented in **Table 4**. Statistical analysis revealed a significant difference between tested salinity levels and organic amendments. In general, the salinity effect was not consistent from one parameter to another. For instance, an increase in salinity level resulted in a reduction of the most investigated parameters, while salinity did not significantly affect root length, root volume, and the number of panicles per plant. For some parameters such as panicle width, panicle dry weight, leaves dry weight, and plant dry weight, the highest values were recorded under the highest salinity level (20 dS·m^-1^). However, organic amendments effects vary across salinity levels. On average, the highest values of different investigated parameters are recorded under goat manure (PH, PnDW, LDW, and PDW), chicken manure (No.Pn, StDia, RV, RDW, StDW), or Lombricompst for panicle width. Days to flowering were significantly increased with increasing salinity. Under high salinity levels, compost and chicken manure recorded the highest no. of days to flowering (44 days), while sheep manure recorded the lowest (37) days.

**Table 4:** Variation of different monitored quinoa morphological traits as affected by salinity level and organic amendments.

Treatments	PH (cm)	No.IN	PnL (cm)	RL (cm)	PnWd (cm)	No.Pn	StDia (mm)	RV (cm^3^)	RDW (g)	StDW (g)	PnDW (g)	LDW (g)	PDW (g)	DtoF (days)
ECiw = 4 dS/m	49.1 ± 7.1 A	28.13 ± 2.34 A	13.99 ± 2.35 AB	17.32 ± 3.02	4.88 ± 1.07 AB	10.47 ± 4.24	6.53 ± 2.1 A	2.57 ± 1.33	1.07 ± 0.54 A	2.11 ± 1.18 A	6.6 ± 3.16 B	3.98 ± 2.16 B	15.05 ± 7.08 AB	38.43 ± 2.87 B
Control	53.67 ± 3.06 ab	27.67 ± 0.58 abcd	14.67 ± 1.16 abc	15 ± 2.65 cde	4.43 ± 0.51 ab	7.67 ± 3.21 b	6.67 ± 1.53 ab	2 ± 1 bc	0.7 ± 0.32 bc	1.34 ± 0.61 bc	5 ± 1.81 ab	3.05 ± 1.33 c	13.79 ± 4.78 bc	41 ± 0 ab
Biochar	45.67 ± 2.89 b	26.67 ± 0.58 cd	11.43 ± 1.6 c	13.5 ± 1.5 e	3.3 ± 0.7 b	7.33 ± 2.52 b	3.67 ± 1.53 b	1.33 ± 0.58 c	0.35 ± 0.1 c	1.03 ± 0.37 c	4.02 ± 2.21 b	2.72 ± 0.88 c	8.12 ± 2.65 c	40.67 ± 4.62 ab
Chicken manure	54.33 ± 3.79 ab	28.33 ± 1.53 abc	14.67 ± 1.53 abc	18.17 ± 1.44 bcd	6.07 ± 0.7 a	20 ± 4.36 a	10 ± 2 a	5 ± 1 a	2.25 ± 0.5 a	4.99 ± 1.04 a	7.8 ± 4.29 ab	7.04 ± 2.26 ab	27.51 ± 5.82 a	38 ± 0 ab
Compost	41.33 ± 6.43 b	24 ± 1 d	16.33 ± 0.58 ab	17.33 ± 1.04 bcde	3.6 ± 0.96 b	8 ± 1 b	6.67 ± 1.53 ab	3.33 ± 1.16 abc	1.07 ± 0.47 bc	1.69 ± 0.71 bc	3.7 ± 1.66 b	2.44 ± 1.46 c	8.01 ± 4.43 c	37 ± 1.73 ab
Cow manure	42.33 ± 5.03 b	31 ± 2 ab	14.33 ± 0.58 bc	18.67 ± 0.58 abc	5.2 ± 0.76 ab	10.67 ± 2.08 b	7 ± 1 ab	2.33 ± 0.58 bc	1.06 ± 0.18 bc	1.82 ± 0.56 bc	8.5 ± 2.25 ab	3.11 ± 1.55 c	17.14 ± 5.85 abc	38 ± 3 ab
Goat manure	61.33 ± 4.62 a	29.67 ± 2.08 abc	13 ± 0 bc	20.17 ± 1.26 ab	5.8 ± 0.99 a	8.67 ± 2.08 b	7 ± 1 ab	1.67 ± 0.58 c	0.98 ± 0.42 bc	1.77 ± 0.34 bc	5.07 ± 1.41 ab	3.03 ± 0.63 c	10.47 ± 2.14 c	37 ± 1.73 ab
Lombricompost	50.67 ± 7.57 ab	27.67 ± 0.58 abcd	12 ± 1 c	14 ± 1 de	4.63 ± 0.32 ab	13 ± 2 ab	5.33 ± 1.53 ab	1.33 ± 0.58 c	0.96 ± 0.12 bc	1.92 ± 0.38 bc	5.81 ± 1.17 ab	2.43 ± 0.36 c	12.5 ± 0.96 bc	42.67 ± 2.89 a
Sheep manure	48 ± 3.46 b	27.67 ± 0.58 abcd	13.33 ± 1.16 bc	15.4 ± 0.53 cde	6 ± 0.5 a	11.33 ± 2.89 b	7 ± 3.61 ab	4 ± 1 ab	1.4 ± 0.38 ab	2.91 ± 0.56 b	11.35 ± 2.58 a	7.88 ± 1.77 a	23.55 ± 5.03 ab	38 ± 0 ab
*H. illucens* frass	50 ± 3 ab	31.33 ± 2.08 a	18.33 ± 2.52 a	18.5 ± 0.5 abc	5.07 ± 0.7 ab	9 ± 3 b	6 ± 1 ab	2.33 ± 0.58 bc	0.93 ± 0.01 bc	2.15 ± 0.29 bc	10.01 ± 2.27 ab	4.52 ± 0.85 abc	17.6 ± 3.41 abc	35 ± 0 b
*T. molitor* frass	43.67 ± 2.89 b	27.33 ± 0.58 bcd	11.83 ± 1.26 c	22.5 ± 2.5 a	4.7 ± 0.3 ab	9 ± 0 b	6 ± 0 ab	2.33 ± 0.58 bc	1.01 ± 0.03 bc	1.48 ± 0.1 bc	4.76 ± 0.82 b	3.61 ± 0.41 bc	11.81 ± 1.97 c	37 ± 1.73 ab
ECiw = 12 dS/m	45.7 ± 8.13 B	27.63 ± 3.22 A	14.68 ± 1.16 A	18.23 ± 3.42	4.54 ± 0.57 B	10.73 ± 3.67	5.69 ± 1.1 B	2.13 ± 0.86	0.84 ± 0.36 B	1.65 ± 0.7 B	6.56 ± 3.02 B	3.37 ± 1.38 B	12.92 ± 5.48 B	39.33 ± 3.76 AB
Control	47 ± 5.2 abc	29.33 ± 2.08 ab	14 ± 1 ab	15.5 ± 1.5 b	4.93 ± 0.12	15 ± 2.65 a	6 ± 1	2.33 ± 0.58 ab	1.03 ± 0.3 abc	2.28 ± 0.64 ab	8.05 ± 1.38 abc	5.35 ± 1.46 a	16.71 ± 3.28 ab	39 ± 1.73 ab
Biochar	42.67 ± 4.04 bc	27.33 ± 1.53 abc	14 ± 1 ab	17.67 ± 0.58 ab	4.57 ± 0.45	11 ± 1 ab	6.67 ± 0.58	2.67 ± 0.58 ab	1 ± 0.18 abc	1.73 ± 0.3 ab	6.33 ± 0.54 bcd	4.42 ± 0.44 ab	14.32 ± 1.75 abc	38 ± 0 ab
Chicken manure	51.33 ± 2.52 ab	25 ± 1 bc	14.33 ± 0.58 ab	20 ± 2 ab	4.57 ± 0.32	11.33 ± 0.58 ab	6 ± 1	2.33 ± 0.58 ab	0.58 ± 0.16 bc	1.24 ± 0.3 b	6.92 ± 1.15 bcd	3.48 ± 0.4 ab	13.59 ± 1.46 abc	37 ± 1.73 b
Compost	43 ± 4.58 abc	26.33 ± 0.58 bc	15 ± 1 ab	16 ± 1 ab	4.63 ± 0.95	12 ± 3 ab	7 ± 2	3.33 ± 1.16 a	1.23 ± 0.47 ab	1.93 ± 0.57 ab	9.63 ± 3.26 ab	3.27 ± 1.33 ab	18.1 ± 6.14 a	37 ± 3.46 b
Cow manure	33.67 ± 3.21 c	27.67 ± 0.58 abc	15.5 ± 0.71 ab	15.33 ± 1.16 b	4.33 ± 0.15	6 ± 0 b	5 ± 1	1.33 ± 0.58 b	0.48 ± 0.03 c	0.92 ± 0.09 b	3.37 ± 1.22 d	2.09 ± 1.03 b	6.86 ± 1.56 c	44.33 ± 2.89 a
Goat manure	40 ± 1.73 bc	22.67 ± 0.58 c	15.67 ± 0.58 ab	21.33 ± 6.29 ab	4.23 ± 0.25	8.67 ± 1.16 ab	5.67 ± 1.16	1.67 ± 1.16 ab	0.48 ± 0.16 c	1.23 ± 0.37 b	3.78 ± 0.52 cd	3.38 ± 0.52 ab	8.08 ± 1.83 abc	44.33 ± 2.89 a
Lombricompost	41 ± 3.61 bc	29 ± 1.73 ab	13.17 ± 1.04 b	16.83 ± 1.26 ab	3.8 ± 0.61	6.67 ± 1.53 b	4 ± 0	1 ± 0 b	0.56 ± 0.01 bc	0.86 ± 0.21 b	2.89 ± 0.8 d	1.56 ± 0.34 b	5.77 ± 1 c	42.67 ± 2.89 ab
Sheep manure	58.33 ± 9.07 a	33 ± 5.57 a	16.25 ± 0.35 a	18.33 ± 1.53 ab	4.37 ± 0.4	8.67 ± 2.31 ab	6.33 ± 1.53	2 ± 0 ab	0.93 ± 0.13 abc	1.74 ± 0.44 ab	5.78 ± 0.84 bcd	3.72 ± 2.16 ab	12.17 ± 2.84 bc	39 ± 3.46 ab
*H. illucens* frass	50.67 ± 10.21 ab	29.33 ± 1.16 ab	14.33 ± 1.53 ab	23.67 ± 4.04 a	5.13 ± 0.85	14 ± 5.29 a	5.67 ± 0.58	2 ± 0 ab	0.8 ± 0.19 abc	1.84 ± 0.97 ab	7.13 ± 1.83 abcd	3.26 ± 0.78 ab	13.03 ± 3.71 abc	36 ± 1.73 b
*T. molitor* frass	49.33 ± 2.08 ab	26.67 ± 0.58 bc	15.33 ± 0.58 ab	17.67 ± 1.53 ab	4.83 ± 0.47	14 ± 2.65 a	5.67 ± 1.16	2.67 ± 0.58 ab	1.3 ± 0.37 a	2.7 ± 0.51 a	11.76 ± 2.31 a	3.2 ± 1.26 ab	20.57 ± 5.64 a	36 ± 1.73 b
ECiw = 20 dS/m	38.53 ± 6.81 C	25.3 ± 2.84 B	13.32 ± 2.41 B	17.87 ± 3.18	4.92 ± 0.97 A	11.28 ± 3.39	5.9 ± 1.4 AB	2.47 ± 1.17	0.95 ± 0.56 AB	1.79 ± 1.09 AB	9.06 ± 4.29 A	4.83 ± 2.04 A	16.36 ± 7.29 A	40.17 ± 2.83 A
Control	30 ± 3.61 b	25.67 ± 0.58 ab	11.33 ± 1.53 b	18.67 ± 2.08 ab	4.53 ± 0.4 bcde	7.67 ± 2.31 c	4.67 ± 0.58 cd	1.67 ± 0.58 bc	0.4 ± 0.18 b	0.98 ± 0.55 d	9.25 ± 1.66 abc	4.54 ± 1.31 bcd	16.29 ± 3.99 abc	41 ± 0 abc
Biochar	35 ± 5.57 ab	25.67 ± 2.31 ab	12.5 ± 0.87 ab	17 ± 1 abc	5.67 ± 0.76 abc	12.67 ± 0.58 abc	7 ± 0 ab	2.67 ± 0.58 abc	1.42 ± 0.49 a	1.39 ± 0.55 cd	9.04 ± 0.41 abc	6.02 ± 1.99 abc	18.67 ± 2.97 abc	41 ± 0 abc
Chicken manure	30.67 ± 0.58 b	24.33 ± 0.58 ab	12 ± 1 ab	13.83 ± 0.29 bc	3.33 ± 0.35 e	7 ± 0 c	3.67 ± 0.58 d	1 ± 0 c	0.24 ± 0.05 b	0.63 ± 0.07 d	2.69 ± 1.26 c	1.96 ± 0.06 d	7.03 ± 1.71 c	44.33 ± 2.89 a
Compost	37.33 ± 1.16 ab	26 ± 2.65 ab	11.67 ± 2.52 ab	19 ± 1 ab	4.17 ± 0.29 de	9 ± 0 bc	6 ± 1 abc	2.33 ± 0.58 bc	1.01 ± 0.17 ab	1.31 ± 0.22 d	6.82 ± 2.16 abc	3.59 ± 0.82 bcd	10.28 ± 1.02 bc	43.33 ± 4.62 ab
Cow manure	38 ± 6.56 ab	21.33 ± 3.06 b	13.33 ± 1.53 ab	17 ± 1.73 abc	4.67 ± 0.61 bcde	12 ± 4.36 abc	6 ± 0 abc	2.33 ± 0.58 bc	0.97 ± 0.41 ab	1.26 ± 0.28 d	8.09 ± 3.48 abc	4.25 ± 0.9 bcd	14.56 ± 3.86 abc	38 ± 0 bc
Goat manure	38.33 ± 4.73 ab	25 ± 2.65 ab	13.33 ± 3.51 ab	21 ± 1 a	5.43 ± 0.81 abcd	16.33 ± 3.79 a	6.67 ± 1.16 abc	4.67 ± 1.53 a	1.76 ± 0.64 a	2.91 ± 1.16 abc	14.43 ± 6.06 a	8.15 ± 1.66 a	27.24 ± 9.41 a	41 ± 0 abc
Lombricompost	42.67 ± 5.69 ab	24 ± 2 ab	16 ± 3 ab	20.33 ± 3.21 a	6.33 ± 0.64 a	11.67 ± 0.58 abc	7.33 ± 0.58 ab	2.67 ± 0.58 abc	1.24 ± 0.33 ab	2.94 ± 0.65 ab	12.99 ± 3.29 ab	6.16 ± 1.41 ab	23.77 ± 3.53 a	38 ± 0 bc
Sheep manure	44.33 ± 5.51 ab	25.33 ± 1.16 ab	12 ± 1 ab	12.73 ± 1.1 c	4.27 ± 0.45 cde	11.33 ± 2.08 abc	5.33 ± 0.58 bcd	1.33 ± 0.58 bc	0.31 ± 0.07 b	0.9 ± 0.23 d	5.12 ± 0.99 bc	2.58 ± 0.24 cd	8.91 ± 1.23 bc	37 ± 1.73 bc
*H. illucens* frass	42 ± 6.08 ab	25.33 ± 1.16 ab	14 ± 0 ab	17.83 ± 2.25 abc	5 ± 0 abcd	14.67 ± 0.58 ab	4.67 ± 1.16 cd	2.67 ± 0.58 abc	1.08 ± 0.41 ab	1.89 ± 0.41 bcd	11.92 ± 3.35 ab	5.26 ± 1.15 abcd	16.71 ± 5.55 abc	40 ± 1.73 abc
*T. molitor* frass	47 ± 6.08 a	30.33 ± 3.79 a	17 ± 1 a	21.33 ± 2.89 a	5.83 ± 0.15 ab	10 ± 1.41 abc	7.67 ± 0.58 a	3.33 ± 0.58 ab	1.12 ± 0.26 ab	3.68 ± 0.27 a	10.32 ± 3.39 abc	5.76 ± 1.12 abc	20.17 ± 5.39 ab	38 ± 0 c

PH, plant height; No.IN, number of internodes; PnL, panicle length; RL, root length; PnWd, panicle width; No.P, number of panicles per plant; StDia, main stem diameter; RV, root volume; RDW, root dry weight; StDW, stem dry weight; PnDW, panicle dry weight; LDW, leaves dry weight; StDW, shoot dry weight; PDW, plant dry weight; Dto, days to flowering. Different letters indicate a significant difference between salinity levels (uppercase letters) and between organic amendments (lowercase letters) at *p* < 0.05 level of significance using the Tukey *post-hoc* test. ECiw, Electrical conductivity of irrigation water.

### 3.2 Crop productivity

The analysis of variance revealed that all investigated productivity parameters are affected (*p*<0.05) by salinity and organic amendments (**Table 5**). Salinity had a tremendous negative impact on biomass and seed yield, with an average reduction of 21 and 36% for fresh biomass, 25 and 37% for dry biomass, 31 and 52% for seed yield, and 29 and 51% for crop water productivity (CWP) under 12 and 20 dS·m^-1^ salinity levels, respectively compared to low salinity level (4 dS·m^-1^). Similarly, harvest index was significantly affected (*p*<0.05) at the high saline condition, with a decrease of 17% compared to the low salinity (4 dS·m^-1^). Regarding organic amendments, their response was inconsistent between one salinity level to another. For instance, averaged across all salinity levels, the insect frass of *Hermetia illucens* recorded the highest value in fresh biomass. In contrast, the *Tenebrio molitor* frass recorded the highest values of dry biomass and seed yield under high salinity. However, under high salinity conditions (20 dS·m^-1^), sheep manure recorded the highest values for dry biomass (1.91 t·ha^-1^), seed yield (1.42 t·ha^-1^), and crop water productivity (0.59 Kg·m^-3^).

**Table 5:** Variation of quinoa fresh and dry biomass yield, seed yield, harvest index (HI), and crop water productivity (CWP) as affected by both salinity and organic amendments.

Treatments	Fresh biomass (t·ha^-1^)	Dry biomass (t·ha^-1^)	Seed yield (t·ha^-1^)	Harvest index (%)	CWP (Kg·m^-3^)
ECiw = 4 dS·m^-1^	10.2 ± 2.77 A	2.22 ± 0.63 A	1.47 ± 0.48 A	36.81 ± 6.94 A	0.59 ± 0.19 A
Control	9.01 ± 1.17 cd	1.7 ± 0.3 cd	1.43 ± 0.31 abc	37.76 ± 4.17 abc	0.59 ± 0.13 abc
Biochar	13.96 ± 2.27 ab	3.39 ± 0.49 a	1.22 ± 0.14 abc	23.85 ± 0.01 c	0.5 ± 0.06 bc
Chicken manure	8.92 ± 2.5 cd	2.04 ± 0.6 bcd	2.04 ± 0.43 a	36.26 ± 6.54 abc	0.84 ± 0.18 a
Compost	9.25 ± 1.02 cd	2.29 ± 0.26 bcd	1.73 ± 0.31 ab	40.58 ± 1.83 abc	0.6 ± 0.08 abc
Cow manure	9.57 ± 1.4 bcd	1.92 ± 0.21 bcd	1.53 ± 0.32 abc	42.5 ± 6.17 ab	0.63 ± 0.13 ab
Goat manure	9.69 ± 1.66 bcd	1.88 ± 0.43 bcd	1.33 ± 0.09 abc	39.73 ± 7.43 abc	0.55 ± 0.04 abc
Lombricompost	6.49 ± 1.3 d	1.58 ± 0.42 d	0.69 ± 0.3 c	28.57 ± 1.65 bc	0.29 ± 0.13 c
Sheep manure	8.57 ± 0.71 cd	1.91 ± 0.18 bcd	1.13 ± 0.4 bc	36.61 ± 7.88 abc	0.42 ± 0.09 bc
*H. illucens* frass	14.59 ± 1.31 a	2.79 ± 0.15 ab	1.49 ± 0.14 abc	33.49 ± 3.53 abc	0.61 ± 0.06 abc
*T. molitor* frass	11.91 ± 1.74 abc	2.67 ± 0.37 abc	2.1 ± 0.44 a	43.84 ± 4.19 a	0.87 ± 0.18 a
ECiw = 12 dS·m^-1^	8.11 ± 1.75 B	1.66 ± 0.43 B	1.01 ± 0.45 B	37.58 ± 8.25 A	0.42 ± 0.18 B
Control	6.97 ± 0.71 bc	1.39 ± 0.23 b	1.08 ± 0.13 bc	35.77 ± 8.33 abc	0.45 ± 0.06 bc
Biochar	7.14 ± 0.37 bc	1.41 ± 0.03 b	1.12 ± 0.05 abc	44.26 ± 0.91 ab	0.46 ± 0.02 abc
Chicken manure	6.94 ± 0.98 bc	1.35 ± 0.17 b	0.8 ± 0.14 c	39.23 ± 0.35 abc	0.33 ± 0.06 c
Compost	8.63 ± 0.8 abc	1.83 ± 0.36 ab	0.97 ± 0.27 c	29.22 ± 3.21 bc	0.4 ± 0.11 c
Cow manure	8.48 ± 0.68 abc	1.77 ± 0.06 ab	1.75 ± 0.2 a	49.63 ± 3.3 a	0.73 ± 0.08 a
Goat manure	7.79 ± 1.07 abc	1.81 ± 0.25 ab	0.68 ± 0.11 c	41.05 ± 11.47 abc	0.28 ± 0.05 c
Lombricompost	5.47 ± 0.46 c	1.1 ± 0.11 b	0.63 ± 0.15 c	36.2 ± 4.21 abc	0.26 ± 0.06 c
Sheep manure	10.57 ± 2.52 a	1.65 ± 0.69 ab	0.7 ± 0.56 c	33.06 ± 8.99 bc	0.29 ± 0.23 c
*H. illucens* frass	9.03 ± 0.67 ab	1.87 ± 0.13 ab	0.62 ± 0.05 c	26.3 ± 2.11 c	0.26 ± 0.02 c
*T. molitor* frass	10.03 ± 1.05 ab	2.42 ± 0.17 a	1.69 ± 0.11 ab	41.08 ± 0.75 abc	0.7 ± 0.05 ab
ECiw = 20 dS·m^-1^	6.51 ± 1.8 C	1.41 ± 0.42 C	0.71 ± 0.36 C	30.36 ± 5.42 B	0.29 ± 0.15 C
Control	5.62 ± 0.08 c	0.87 ± 0.55 b	0.53 ± 0.2 b	26.87 ± 2.49 de	0.22 ± 0.08 b
Biochar	5.86 ± 0.37 bc	1.08 ± 0.25 ab	0.47 ± 0.19 b	23.74 ± 0.78 de	0.2 ± 0.08 b
Chicken manure	5.81 ± 0.77 c	1.07 ± 0.02 ab	0.53 ± 0.03 b	33.03 ± 1.14 bc	0.22 ± 0.02 b
Compost	8.74 ± 0.74 a	1.78 ± 0.59 ab	0.51 ± 0.19 b	29.54 ± 1.37 cde	0.21 ± 0.08 b
Cow manure	6 ± 0.28 bc	1.4 ± 0.2 ab	0.67 ± 0.19 ab	34.63 ± 0.13 abc	0.28 ± 0.08 ab
Goat manure	5.88 ± 1.13 bc	1.37 ± 0.29 ab	0.68 ± 0.13 ab	38.42 ± 3.42 ab	0.28 ± 0.05 ab
Lombricompost	7.64 ± 0.54 abc	1.43 ± 0.13 ab	0.57 ± 0.06 b	28.58 ± 1.93 cde	0.24 ± 0.02 b
Sheep manure	6.88 ± 0.23 abc	1.91 ± 0.42 a	1.42 ± 0.54 a	31.74 ± 2.45 bcd	0.59 ± 0.22 a
*H. illucens* frass	7.8 ± 0.66 ab	1.73 ± 0.01 ab	0.97 ± 0.38 ab	40.7 ± 1.48 a	0.4 ± 0.16 ab
*T. molitor* frass	6.13 ± 0.01 bc	1.44 ± 0.26 ab	0.74 ± 0.4 ab	26.31 ± 2.31 de	0.31 ± 0.17 ab

Different letters indicate a significant difference between salinity levels (uppercase letters) and between organic amendments (lowercase letters) at *p* < 0.05 level of significance using the Tukey *post-hoc* test. ECiw, Electrical conductivity of irrigation water.

### 3.3 Soil salinity and humidity

**Figure 3:**
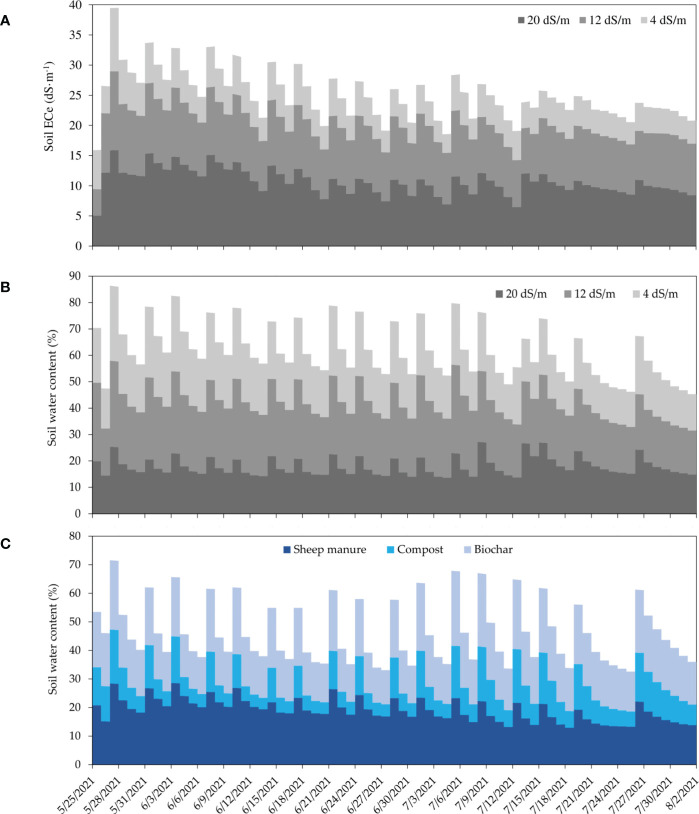
Stacked area plot of daily soil salinity (ECe) dynamics during the crop growing period measured under sheep manure amendment and three irrigation water salinity levels at 0-10 cm soil depth **(A)**. Stacked area plot of daily soil moisture dynamics during the crop growing period measured under sheep manure amendment and three irrigation water salinity levels at 0-10 cm soil depth. **(B)**, and under three organic amendments (sheep manure, compost, and biochar) measured at irrigation water salinity of (4 dS·m-1) **(C)**, at 0-15 cm soil depth.

Results of the soil salinity dynamics under sheep manure and different salinity levels are shown as stacked area plot in **Figure 3A**. The obtained data indicated that the measured soil ECe is well correlated with the applied salt concentration and varied according to soil moisture, as the soil EC sensors are very sensitive to soil humidity. The daily average of ECe varies from 5 to 15 dS·m^-1^, from 4 to 12 dS·m^-1^, and from 3 to 8 dS·m^-1^, under 4, 12, and 20 dS·m^-1^ of irrigation water salinity, respectively.

Soil moisture was less affected by salinity, and the average daily soil moisture were 19, 24, and 18% under low, medium, and high salinity levels, respectively (**Figure 3B**). Variations of soil moisture under low salinity level (4 dS·m^-1^) and three different organic amendments are shown in **Figure 3C**. Measured data clearly indicate that soil moisture varied from one amendment to another. For instance, biochar applied with 5 t·ha^-1^ holds more water than the compost applied with the same rate. Moreover, it holds more or less the same moisture (20%) as sheep manure applied at a rate of 30 t·ha^-1^.

### 3.4 Chlorophyll content

**Figure 4:**
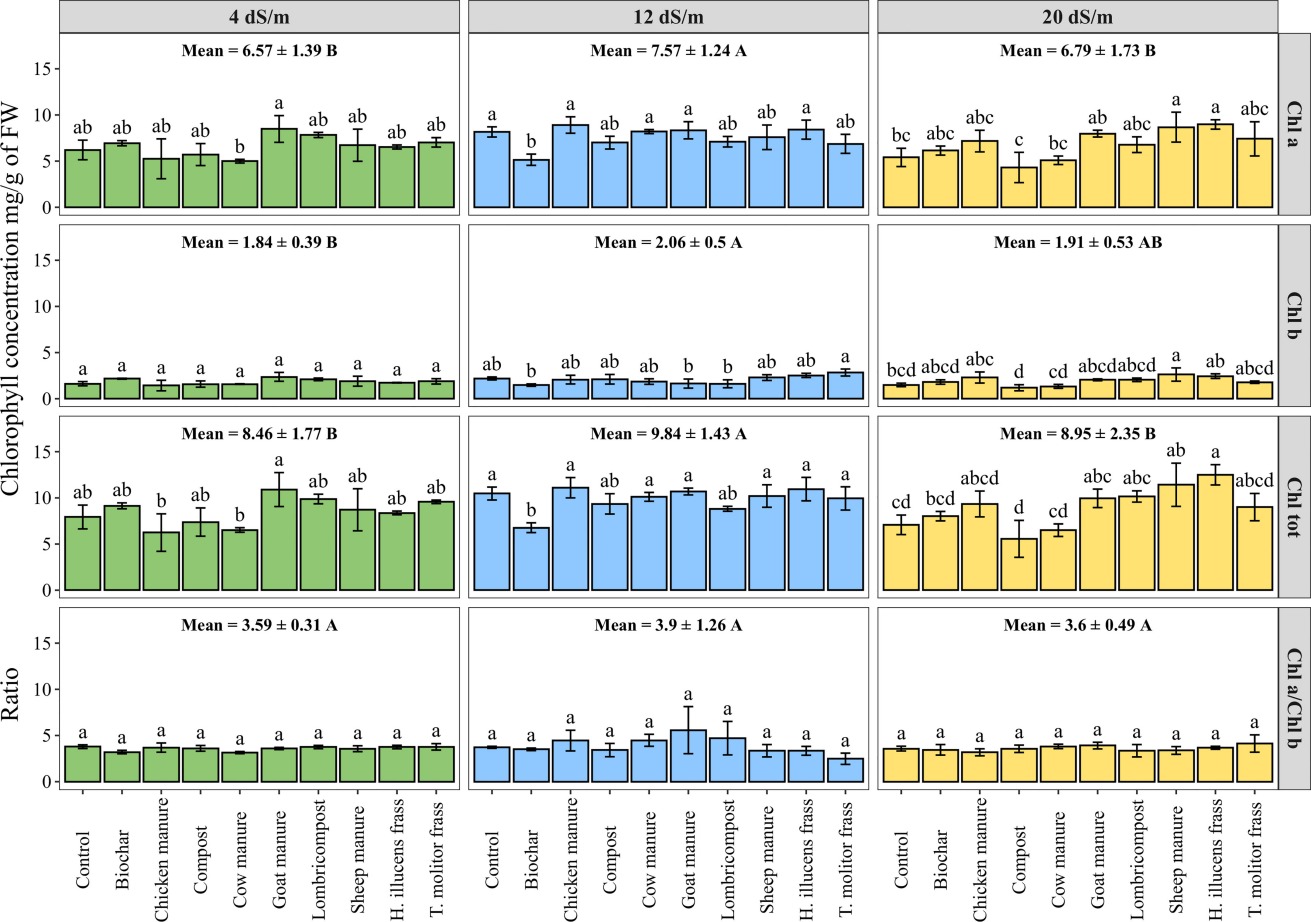
Photosynthetic pigments content measured on fresh leaves of quinoa as affected by different irrigation water salinity and organic amendments. Chlorophyll a (Chl a), chlorophyll b (Chl b), total chlorophyll (Chl tot), and chlorophyll a to chlorophyll b ratio (Chl a/Chl b). Values represent mean ± standard deviation. Different letters indicate a significant difference between salinity levels (uppercase letters) and between organic amendments (lowercase letters) at *p* < 0.05 level of significance using the Tukey *post-hoc* test.

Chlorophyll in leaves is essential for plant growth and can be used to determine a plant’s reaction to stress. The analysis of variance was conducted to assess the effect of irrigation water salinity on chlorophyll pigments concentration under tested organic amendments (**Figure 4**). Results indicate a significant effect of irrigation water salinity on chlorophyll a (Chl a), chlorophyll b (Chl b), and total chlorophyll (Chl tot) contents. As an average of all tested amendments, the maximum concentration in terms of total chlorophyll was about 9.84 ± 1.43 mg/g of FW under medium salinity (12 dS·m^-1^). However, organic amendments significantly affected the contents of all measured pigments, except for chlorophyll b content under low salinity levels. While the insect frass of *H. illucens* and sheep manure significantly increased the total chlorophyll concentration by 76% and 61%, respectively, compared to the control under high salinity conditions (20 dS·m^-1^). Additionally, no significant difference was recorded for the chlorophyll a to chlorophyll b ratio under salinity conditions and for all tested organic amendments.

### 3.5 Leaf mineral content

Analysis of variance revealed a significant effect (*p* < 0.01) of salinity and organic amendments on sodium content of quinoa. Conversely, the potassium content was not affected by the salinity treatments, and the effects of the organic amendments were only noticeable in the medium and high salinity conditions, which is demonstrated in **Table 6**. Results indicate an increase of 75% in sodium accumulation under high salinity (20 dS/m) compared to low salinity conditions (4 dS/m). In terms of amendment, goat manure was found to decrease sodium accumulation on the leaves of quinoa with 0.85 and 1.45 g/100g DW, under low and medium irrigation water salinity (12 dS/m), respectively. However, under high salinity conditions, sheep manure was more efficient in decreasing the sodium content (1.66 g/100g DW), increasing the potassium accumulation (12.53 g/100g DW), and subsequently resulting in a higher K/Na ratio (7.88).

**Table 6:** Sodium and potassium contents, and K/Na ratio in quinoa leaves as affected by different salinity levels and various organic amendments.

Treatments	Na (g/100 g DW)	K (g/100 g DW)	K/Na ratio
ECiw = 4 dS·m^-1^	1.3 ± 0.07 C	9.77 ± 0.2	8.34 ± 0.6 A
Control	1.27 ± 0.07 abc	9 ± 0.48	7.13 ± 0.4
Biochar	0.97 ± 0.2 c	10.6 ± 0.23	11.63 ± 1.8
Chicken manure	1.17 ± 0.14 bc	10.16 ± 0.53	8.97 ± 1.14
Compost	1.21 ± 0.24 abc	9.67 ± 0.83	9.16 ± 2.91
Cow manure	1.72 ± 0.23 a	9.04 ± 0.7	5.5 ± 1.08
Goat manure	0.85 ± 0.12 c	9.91 ± 0.69	12.23 ± 2.01
Lombricompost	1.59 ± 0.26 ab	9.65 ± 0.48	6.58 ± 1.62
Sheep manure	1.31 ± 0.28 abc	10.84 ± 0.5	9.26 ± 2.45
*H. illucens* frass	1.26 ± 0.03 abc	8.81 ± 0.34	7.03 ± 0.45
*T. molitor* frass	1.68 ± 0.03 ab	10.02 ± 0.91	5.95 ± 0.44
ECiw = 12 dS·m^-1^	1.96 ± 0.08 B	9.74 ± 0.16	5.23 ± 0.25 B
Control	1.51 ± 0.03 d	10.1 ± 0.23 abc	6.7 ± 0.18 ab
Biochar	2 ± 0.12 abcd	8.55 ± 0.35 c	4.32 ± 0.34 bc
Chicken manure	1.91 ± 0.05 abcd	9.8 ± 0.06 abc	5.15 ± 0.17 bc
Compost	1.92 ± 0.18 abcd	9.63 ± 0.3 abc	5.14 ± 0.64 bc
Cow manure	2.32 ± 0.18 abc	9.06 ± 0.43 bc	3.98 ± 0.5 c
Goat manure	1.45 ± 0.13 d	10.81 ± 0.24 a	7.62 ± 0.79 a
Lombricompost	2.41 ± 0.01 ab	10.06 ± 0.32 abc	4.18 ± 0.16 c
Sheep manure	1.86 ± 0.22 bcd	8.84 ± 0.52 c	4.94 ± 0.83 bc
*H. illucens* frass	2.57 ± 0.19 a	10.61 ± 0.29 ab	4.16 ± 0.21 c
*T. molitor* frass	1.63 ± 0.08 cd	9.93 ± 0.46 abc	6.11 ± 0.26 abc
ECiw = 20 dS·m^-1^	2.29 ± 0.11 A	9.29 ± 0.29	4.47 ± 0.34 B
Control	2.14 ± 0.3 ab	8.79 ± 0.17 b	4.27 ± 0.62 ab
Biochar	2.26 ± 0.35 ab	9.62 ± 0.82 ab	4.55 ± 0.95 ab
Chicken manure	1.55 ± 0.25 b	9.21 ± 0.97 ab	6.31 ± 1.45 ab
Compost	3.19 ± 0.24 a	9.24 ± 0.9 ab	2.94 ± 0.39 b
Cow manure	2.42 ± 0.25 ab	9.47 ± 0.9 ab	4.06 ± 0.72 ab
Goat manure	2.64 ± 0.22 ab	8.19 ± 1.01 b	3.2 ± 0.59 b
Lombricompost	2.06 ± 0.25 ab	9 ± 0.31 ab	4.46 ± 0.37 ab
Sheep manure	1.66 ± 0.25 b	12.53 ± 0.59 a	7.88 ± 1.12 a
*H. illucens* frass	2.15 ± 0.13 ab	8.24 ± 0.6 b	3.85 ± 0.26 b
*T. molitor* frass	2.84 ± 0.39 ab	8.62 ± 0.2 b	3.13 ± 0.35 b

Values represent mean ± standard deviation. Different letters indicate a significant difference between salinity at p < 0.05 using the Tukey *post-hoc* test.

#ECiw, Electrical conductivity of irrigation water.

### 3.6 Leaf proline content

**Figure 5:**
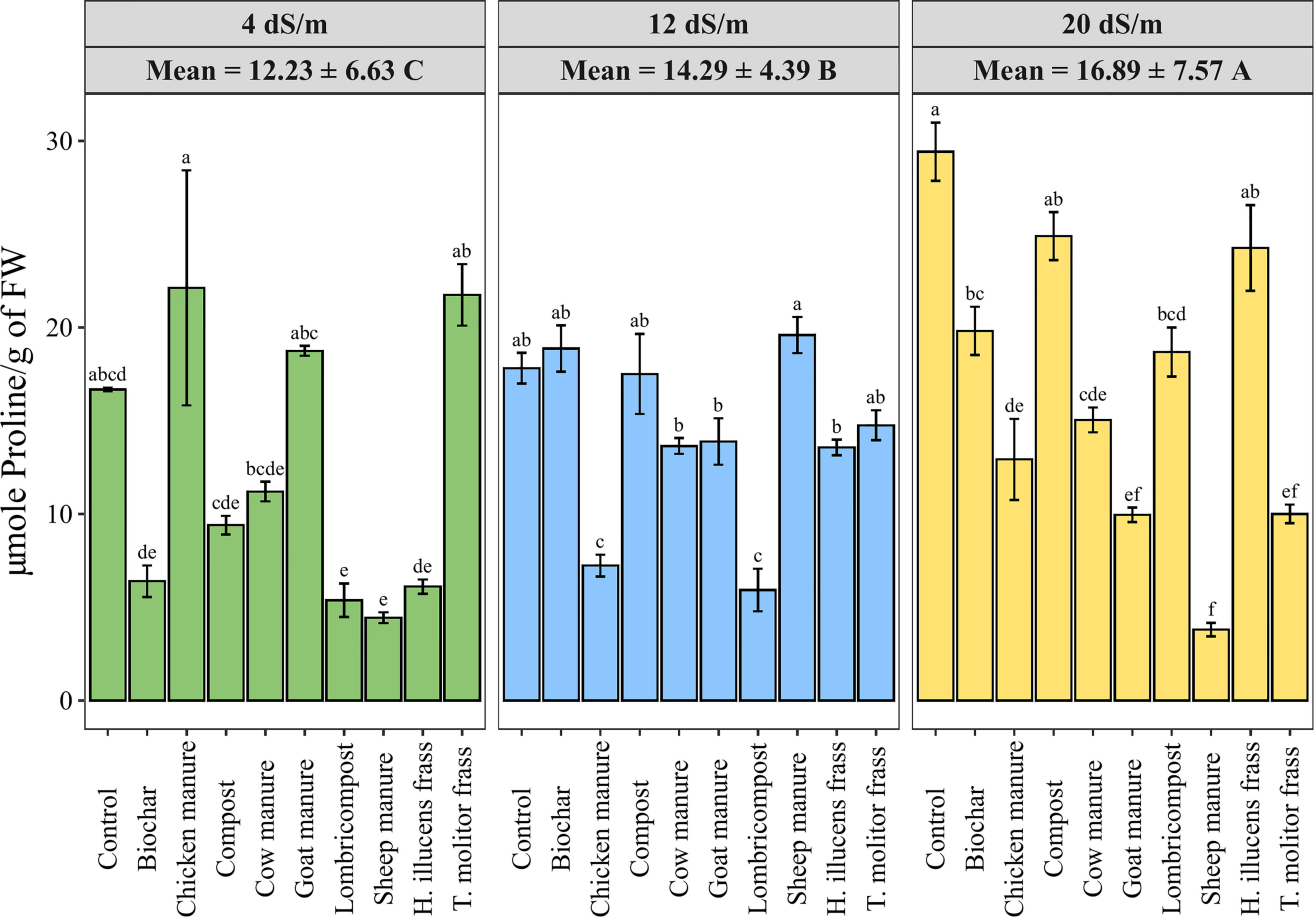
Variation of quinoa’s leaf Proline content under different salinity levels and various organic amendments. Values represent mean ± standard deviation. Different letters indicate a significant difference between salinity levels (uppercase letters) and between organic amendments (lowercase letters) at *p* < 0.05 level of significance using the Tukey *post-hoc* test.

Accumulating endogenous osmolytes in a plant’s leaf is a valuable indicator of the tolerance to osmotic stress. Free proline content measured under different irrigation water salinity levels and various organic amendments in quinoa leaves is shown in **Figure 5**. Data showed a significant increase of proline content by 38% from low to high salinity levels. However, amendments significantly decreased the proline level compared to the control at high salinity conditions. Meanwhile, Compost exhibited the highest proline content (24.9 µmole of Proline/g of FW), while sheep manure showed the lowest value (3.81 µmole of Proline/g of FW).

### 3.7 Seed saponin content

**Figure 6:**
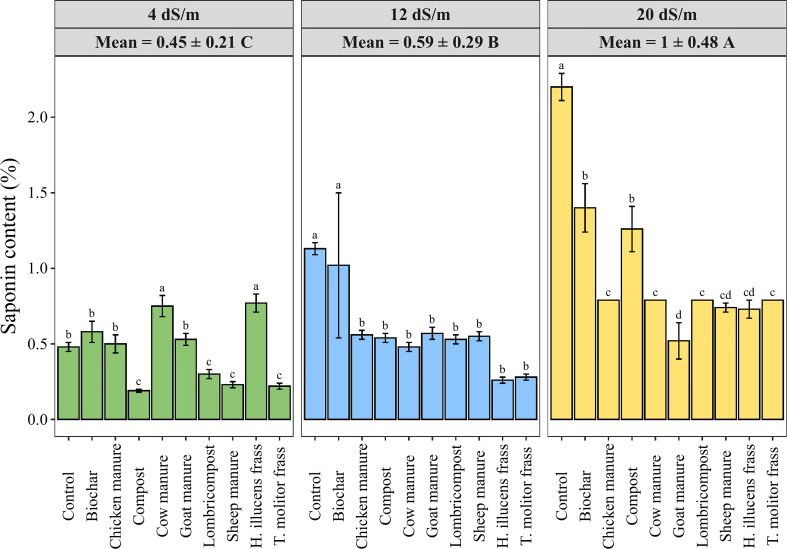
Variation of seed saponin content in quinoa as affected by different irrigation water salinity levels and organic amendments. Values represent mean ± standard deviation. Different letters indicate a significant difference between salinity levels (uppercase letters) and between organic amendments (lowercase letters) at *p* < 0.05 level of significance using the Tukey *post-hoc* test.

Saponins are a class of compounds found in a wide variety of plants and are known for their characteristic bitter taste. The saponins content was measured in quinoa seeds in response to various organic amendments under three different salinity levels. Results presented in **Figure 6** showed a significant increase in saponins content when salinity increased in different amendments averaging values of 0.45 ± 0.21, 0.59 ± 0.29, 1 ± 0.48% of DW under salinity levels of 4, 12, and 20 dS·m^-1^, respectively. Meanwhile, applying organic amendments significantly decreased the seed’s saponin content compared to the control in medium and high salinity levels. Hence, the application of goat manure decreased the saponin content by 76% at high salinity conditions compared to the control treatment.

### 3.8 Seed polyphenol content

**Figure 7:**
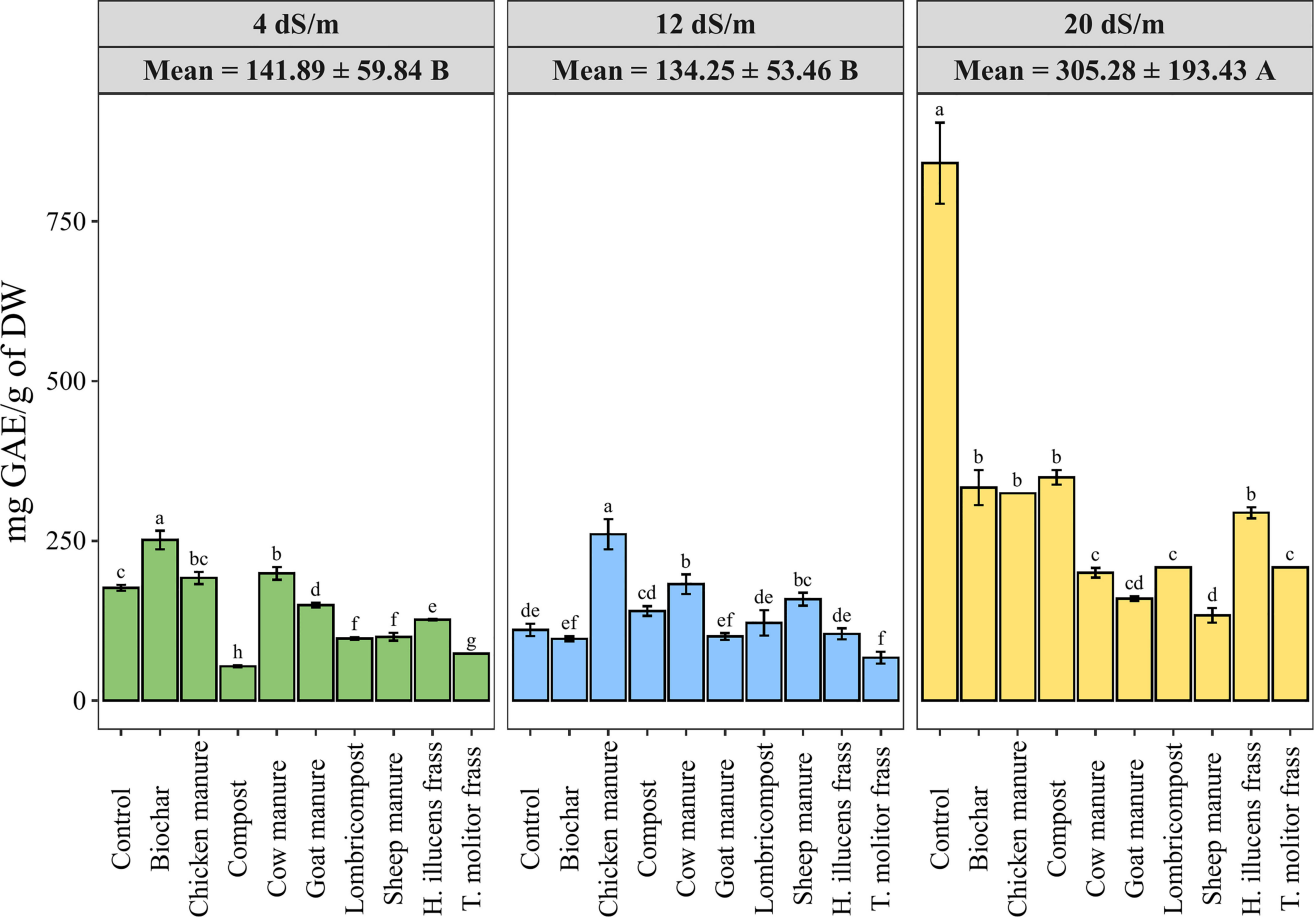
Variation of quinoa phenolic content under different salinity levels and various organic amendments. Values represent mean ± standard deviation. Different letters indicate a significant difference between salinity levels (uppercase letters) and between organic amendments (lowercase letters) at *p* < 0.05 level of significance using the Tukey *post-hoc* test.

Polyphenols are a large group of naturally occurring compounds that are known for their antioxidant properties and are found in a wide variety of plants. The effects of salinity level and organic amendments on the polyphenols content (TPC) in quinoa seeds are presented in **Figure 7**. Data showed a significant effect of salinity on the production of polyphenolic compounds. Hence, higher salinity levels led to higher phenolic content in different organic amendments. Furthermore, applying different amendment treatments significantly decreased the total phenolic content in quinoa seeds compared to the untreated group. The use of sheep manure recorded the lowest value of TPC under a highly saline irrigation supply (133.52 mg GAE/g of DW). At the same time, the compost significantly increases the TPC in quinoa seed from 53.55 ± 1.23 to 349.48 ± 7.99 mg GAE/100 g of DW under 4 and 20 dS·m^-1^, respectively.

### 3.9 Seed antioxidant activity

Plants’ antioxidant mechanism is triggered in response to the abiotic stress caused by excess salt exposure. This mechanism involves the activation of various enzymatic and non-enzymatic antioxidants that work to scavenge reactive oxygen species (ROS) produced under abiotic stress. The antioxidant capacity of quinoa seed is measured by DPPH, ABTS, and ferric-reducing antioxidant capacity. Quinoa seeds exhibited good antioxidant activity (AOX) across different groups of amendments, as depicted in **Table 7**. Compared to the control group, all amendment groups showed higher AOX when measured using the ABTS and FRAP assays, although there was no significant variation in antioxidant activity when measured using the DPPH assay. At ECiw = 4 dS/m, the highest AOX value was recorded in *T. molitor* frass using the DPPH assay (147.45 ± 1.15 µmol TE/g E), followed by *H. illucens* frass (147.22 ± 1.7 µmol TE/g E). Goat manure exhibited the highest FRAP value of 11.13 ± 0.99 µmol TE/g DW at ECiw = 20 dS/m, while compost showed the maximum value of 34.78 ± 2.3 µmol TE/g DW when measured using the ABTS assay at ECiw = 20 dS/m.

**Table 7:** Antioxidant content in quinoa as affected by different salinity levels and various organic amendments.

Treatments	DPPH µmol TE/g E	FRAP µmol TE/g DW	ABTS µmol TE/g E
ECiw = 4 dS·m^-1^	110.98 ± 24.38 A	7.35 ± 0.41 B	21.5 ± 1.56 C
Control	92.01 ± 2.23 e	8.2 ± 0.17 ab	8.01 ± 0.92 f
Biochar	79.34 ± 1.27 f	9.26 ± 0.82 a	27.55 ± 3.06 ab
Chicken manure	129.53 ± 1.27 b	5.85 ± 0.36 ab	27.9 ± 0.74 ab
Compost	106.28 ± 2.98 d	4.04 ± 0.26 b	13.44 ± 1.24 ef
Cow manure	118.35 ± 1.06 c	8.72 ± 1.11 a	30.95 ± 1.36 ab
Goat manure	92.01 ± 1.2 e	9.38 ± 0.66 a	15.33 ± 0.57 de
Lombricompost	119.33 ± 0.9 c	5.13 ± 0.1 ab	20.41 ± 0.28 cd
Sheep manure	78.26 ± 0.74 f	7.19 ± 1.3 ab	12.88 ± 0.22 ef
*H. illucens* frass	147.22 ± 1.7 a	9.13 ± 0.16 a	32.97 ± 0.49 a
*T. molitor* frass	147.45 ± 1.15 a	6.61 ± 1.98 ab	25.55 ± 0.22 bc
ECiw = 12 dS·m^-1^	104.29 ± 20.09 C	7.46 ± 0.36 B	23.33 ± 1.22 B
Control	78.16 ± 1.91 e	6.09 ± 1.22 bc	23.95 ± 3.97 bc
Biochar	83.92 ± 1.63 de	6.42 ± 0.41 bc	29.4 ± 0.94 ab
Chicken manure	115.87 ± 0.85 b	7.52 ± 0.04 bc	28.71 ± 0.28 ab
Compost	102.12 ± 0.92 c	7.96 ± 0.43 b	11.79 ± 0.79 d
Cow manure	91.55 ± 1.21 d	11.42 ± 1.02 a	29.69 ± 0.39 ab
Goat manure	105.02 ± 0.9 c	8.42 ± 0.94 ab	18.07 ± 1.11 cd
Lombricompost	137.49 ± 0.64 a	7.56 ± 0.16 bc	31.39 ± 0.21 a
Sheep manure	134.87 ± 3.51 a	6.76 ± 0.12 bc	24.22 ± 1.23 abc
*H. illucens* frass	111.38 ± 1.01 bc	7.8 ± 0.34 bc	18.46 ± 0.35 cd
*T. molitor* frass	82.54 ± 9.61 de	4.62 ± 0.34 c	17.6 ± 0.57 cd
ECiw = 20 dS·m^-1^	106.57 ± 26.84 B	10.01 ± 0.24 A	30.33 ± 1.23 A
Control	72.46 ± 2.37 h	10.56 ± 0.43 ab	25.37 ± 2.12 cd
Biochar	81.72 ± 2.55 fg	11.06 ± 0.53 ab	35.26 ± 1.44 ab
Chicken manure	122.14 ± 2.2 c	9.34 ± 0.33 ab	31.63 ± 0.97 bc
Compost	90.61 ± 3.53 ef	9.03 ± 0.76 ab	34.78 ± 2.3 ab
Cow manure	72.98 ± 7.12 gh	11.91 ± 0.65 a	30.88 ± 1.21 bc
Goat manure	106.19 ± 0.28 d	11.13 ± 0.99 ab	29.4 ± 0.89 bc
Lombricompost	146.61 ± 0.42 a	9.57 ± 0.13 ab	42.13 ± 1.8 a
Sheep manure	133.51 ± 1.62 b	8.76 ± 0.36 b	23.72 ± 2.33 cd
*H. illucens* frass	142.68 ± 1.28 a	9.16 ± 0.71 ab	30.74 ± 1.57 bc
*T. molitor* frass	96.79 ± 3.86 e	9.59 ± 0.55 ab	19.37 ± 2.05 d

Values represent mean ± standard deviation. Different letters indicate a significant difference between salinity at p < 0.05 using the Tukey *post-hoc* test.

ECiw, Electrical conductivity of irrigation water.

### 3.10 Correlation matrix and principal component analysis

**Figure 8:**
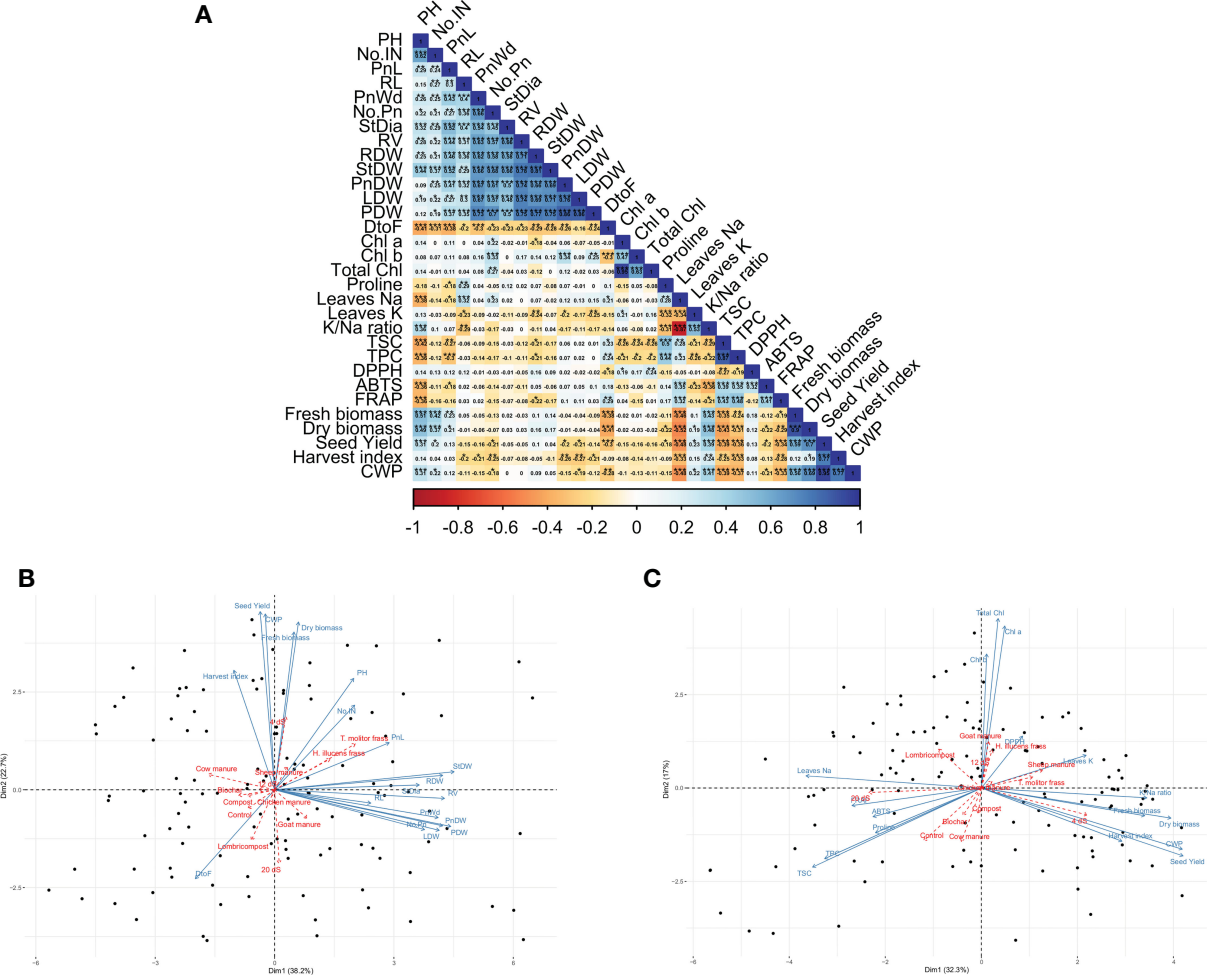
Pearson’s correlation matrix for the investigated parameters. Values in the matrix represent Pearson’s correlation coefficient. *, **, *** indicate the significance of the correlation coefficient at p < 0.05, 0.01, and 0.001, respectively **(A)**. PCA-biplot projection of individuals (black points), variables (blue arrows), and supplementary qualitative variables (red arrows) on the main axis for agro-morphological and productivity traits **(B)**, and biochemical and physiological parameters **(C)**.

**Figure 8A** presents the correlation between the investigated parameters. Results indicate a significant positive correlation among agro-morphological traits. In contrast, days to flowering negatively correlate with almost all agro-morphological and crop productivity parameters. However, total saponin content (TSC), total polyphenol (TPC), and proline content have a significant negative correlation with dry biomass and seed yield.

The principal plan explains 61% of the total variability in agro-morphological and productivity parameters (**Figure 8B**), and 49% for the relationship between productivity traits and biochemical and physiological measurements (**Figure 8C**). Plotting the treatments as a supplementary qualitative variable indicates that the clustering could be revealed, especially for the irrigation water salinity treatment. However, most of the organic amendments are not well presented on the first two dimensions. The results indicate that productivity parameters, such as biomass production and seed yield, are positively correlated with low salinity treatment (4 dS/m). Consequently, the same productivity parameters had a strong negative correlation with high salinity treatment (20 dS/m).

In terms of biochemical composition, the factor analysis revealed a strong positive correlation between TSC, TPC, ABTS, FRAP, Proline, and sodium content with the high salinity treatment (20 dS/m), while low salinity treatment is mainly associated with a high K/Na ratio. Regarding the organic amendments, T. molitor and H. illucens frass are positively correlated with most of the agro-morphological traits, while the non-treated control is positively correlated with TSC and TPC.

## 4 Discussion

It is well known that quinoa is a facultative halophyte that resists to high salinity levels without a notable effect on growth and development (Adolf et al., 2013). In contrast, in our experiment the effect of salinity on different measured morphological traits was pronounced, and a significant effect of salinity was noticed. Our findings are aligned with several previous studies reporting the impact of salinity on quinoa growth and productivity. For instance, Long (2016) found that plant height, root length, shoot dry weight, root dry weight, and panicle length were slightly reduced under high salinity conditions with a reduction rate of 13, 17, 9, 12 and 10% under high salinity level (150 mM) compared to the control (0 mM). Similarly, Talebnejad and Sepaskhah (2015) found that salinity increase from 10 to 20 dS·m^-1^ slightly reduced plant height, shoot and root dry weight by about 3, 10 and 15%, respectively. Contrarily to our results where number of panicles per plant increased by 8% under high salinity, Rezzouk et al. (2020) reported that salinity reduced the number of inflorescence or panicle per plant by 12% under saline compared to non-saline conditions. However, their results in terms of plant height and panicle length are in agreement with the finding of the present study. In relation to the quinoa genotype ICBA-Q5 utilized in our experiment, numerous studies have been carried out in arid regions to evaluate the impact of salinity on quinoa’s morphological characteristics. However, a recent study by Shahid and Thushar (2021) indicated a reduction in plant height, number of panicles, and panicle length by 22%, 8%, and 12%, respectively, when subjected to saline irrigation (ECiw = 15 dS/m) in comparison to freshwater irrigation (ECiw = 0.3 dS/m).

Regarding quinoa productivity including seed and biomass yield, several studies reported a similar findings for other tested varieties. For example, Jacobsen et al. (2003) found that seed yield of quinoa was reduced by 10% under 20 dS·m^-1^ compared to 5 dS·m^-1^, while under fresh water conditions, the quinoa seed yield was similar to that obtained under 20 dS·m^-1^. They also found that the highest seed yield was obtained under 15 dS·m^-1^ and the lowest yield under seawater salinity level (40 dS·m^-1^) with a reduction rate of only 44% compared to the highest yield. Similarly, (Koyro and Eisa, 2008) showed that quinoa seed yield only decreased by about 15 and 16% under 10 and 20 mM NaCl, respectively compared to the control (0 mM NaCl). While quinoa seed yield was greatly declined under 300 and 500 mM NaCl by 44 and 97% respectively, compared to the control. Similarly to our finding (Iftikhar Hussain et al., 2020) reported that salinity has negatively affected quinoa growth and productivity. In fact, seed yield was reduced by 44 and 60% under 10 and 20 dS·m^-1^, respectively compared to the control (0 dS·m^-1^). However, dry biomass yield seemed to be less affected by salinity as it was only reduced by 2 and 3% under 10 and 20 dS·m^-1^ respectively. Contrarily, in a previous experiment in the same experiment site using the same quinoa variety (ICBA-Q5), Bouras et al. (2022) showed that yield was significantly increased by 27 and 13% under saline irrigation water with EC values up to 12 and 17 dS·m^-1^ respectively, compared with the control. Similarly, the same experiment showed an increase in biomass yield by 17 and 29% higher at saline irrigation with EC values of 12 and17 dS·m^-1^, respectively, compared with the control.

All those reported findings showed that quinoa has the ability to resist and produce under high salinity conditions. Quinoa resistance to salinity involves several mechanisms. One of them is its ability to accumulate salt ions in its cells and tissues in order to control and adjust leaf water potential. This enables the plants to maintain cell turgor and therefore, limit transpiration under saline conditions, thus, avoiding physiological damage (Jacobsen et al., 2003). Adolf et al. (2013) summarized the different physiological mechanisms that quinoa deploys to resist to salinity including: (1) stomata regulation and density by maintaining stomatal conductance and reducing stomata density under salinity conditions, (2) exclusion of toxic ions in the salt bladders, (3) osmo-protection by accumulating compatible osmolytes such as proline and mannitol, (4) osmotic adjustment by using inorganic ions such as Na, K and Cl as cheap osmotic in shoot tissue, (5) sodium translocation at xylem parenchyma rather than Na extrusion and (6) potassium retention by increasing K uptake and maintain a high K/Na ratio under salinity conditions. In our experiment, the determination of mineral content indicates a high accumulation of sodium on quinoa leaves under high salinity conditions, resulting in an increase of the K/Na ratio. However, Oumasst et al. (2022) observed a similar trend for sodium accumulation in the ICBA-Q5 variety under arid conditions in the south of Morocco. They found that the sodium content increased tenfold under 10 dS/m of irrigation water salinity compared to 0.9 dS/m salinity level. On the other hand, Bouras et al. (2022) reported no significant effect on the K/Na ratio. However, they found that both sodium and potassium contents increased by 60% and 65%, respectively, under high salinity conditions (17 dS/m) compared to the control (5 dS/m) at the same experimental site using ICBA-Q5 as a quinoa cultivar.

The organic amendment effect on quinoa was reported by several studies to improve its growth and development. Organic amendment mitigates the adverse effects of salinity by several ways including nutrient availability increase, improving soil structure which ease the salt leaching and improve soil aeration, increasing soil water holding capacity, improving soil micro-organisms activity (Diacono and Montemurro, 2015). Thus, plants assimilate more nutrients and water and accumulate more reserves and biomass. The organic amendment can also be beneficial under salinity conditions. Our finding indicates clearly that all tested organic amendments resulted in average more or less similar or higher values of seed and biomass yield compared to the control. Under high salinity conditions (20 dS·m^-1^), most of tested organic amendments have improved quinoa seed yield. For instance, sheep manure and insect frass of *Hermetia illucens* increased seed yield by 167 and 83% compared to the control. Very few reports are available on the impact of organic amendment on quinoa productivity under salinity conditions. Abdrabou et al. (2022) reported that applying 20 t·ha^-1^ farmyard manure to quinoa increased seed yield by 6-fold compared to non-treated control. Alcívar et al. (2018) found that application of biochar, humic substances or both combined significantly improved quinoa root dry weight, root length and seed yield, conversely our study showed that applying biochar at a rate of 5 t·ha^-1^ under high salinity conditions reduced yield by 11% compared to the control. However, under medium salinity level biochar significantly improved yield by 4% compared with the control. Gill et al. (2020) reported that an application of 20 and 30 t·ha^-1^ of biochar resulted in a significant increase in quinoa biomass by 54 and 113%, respectively compared to the control. Similarly, seed yield also increased by 116 and 153%, respectively under 20 and 30 t·ha^-1^ of biochar.

The finding of this study revealed that biochar significantly improved seed yield by 4% under medium salinity (12 dS·m^-1^) level compared with the control, with no improvement under high salinity conditions (20 dS·m^-1^). While compost improved seed yield by 21% under low (4 dS·m^-1^) salinity conditions, while it reduced seed yield by 11% under medium salinity level (12 dS·m^-1^) compared to the control. The finding also indicates that soil moisture under low salinity conditions and biochar application was maintained much higher than compost applied with the same dose. It is more probable that the high-water holding capacity of biochar compared to compost is among the factors that led to an improvement of the quinoa biomass and seed yield, especially under salinity conditions (Yang et al., 2020). While under high salinity conditions, quinoa responded better to compost rather than biochar due to its high carbon to nitrogen ratio compared with biochar in which the organic matter is more stable (**Table 3**).

It is very difficult to explain the quinoa yield improvement under different organic amendments and different salinity levels as the behavior of each amendment varies from salinity level to another. Moreover, the yield improvement can be explained by amendment nutrient content, mineralization rate and speed, ability to improve soil water holding capacity, and biological activity. Several soil physical properties are adversely affected by salinity, making it challenging for plants to establish and thrive. The high concentration of sodium ions in the soil causes dispersion and swelling of aggregates, leading to reduced permeability and increased crusting of the soil surface (Arora and Dagar, 2019). To counter these effects, organic amendments such as manures and composts can be added to increase the concentration of humic colloids in the soil, thereby improving the structural stability of soil aggregates and enhancing water-holding capacity (Dorado et al., 2003). Diacono and Montemurro (2011) have reported that animal manure’s high negative charge increases the organic carbon content, resulting in a greater cation exchange capacity, which helps in reducing nutrient loss. This is important in enhancing the organic carbon content, available phosphorus, and extractable potassium, making them readily available for plant growth. On the other hand, Goss et al. (2013) conducted a review of several contaminants associated with animal manure that can potentially pose health risks and environmental hazards. These contaminants include pathogens such as bacteria, viruses, protozoa, and helminthic worms, as well as macronutrients and trace elements. These contaminants can infect both farm animals and humans through contaminated feed, food, and water supplies, and thus pose potential limitations and challenges associated with the use of organic amendments in large-scale agricultural production. Other limitations that may face farmers using large amounts of organic amendments under salinity is the consequent costs associated to their purchase and application.

Proline is a proteinogenic amino acid that accumulates naturally in plants under various biotic and abiotic stress (Hare and Cress, 1997; El Moukhtari et al., 2020; Pingle et al., 2022). Accumulation of proline in cytosol and plastids of plant tissues engenders the adjustment of cellular osmotic pressure and maintains the plant’s cell turgidity (Szepesi and Szollosi, 2018). In this present study, increased salinity level led to an increasing free proline level in different groups of amendments and control. These results agreed with previous studies reporting that salt stress caused a significant increase of proline accumulation by 5-folds in shoots of Indian green gram (Misra and Gupta, 2005), similarly, (Hmidi et al., 2018) showed that proline content and amino acids increase with increasing salinity and duration treatment. Meanwhile, the application of organic amendments in quinoa significantly increased the proline content. In agreement, previous studies reported the accumulation of osmoprotectants in amendments-treated plants under salt-stress conditions (Flowers and Colmer, 2008; Talaat and Shawky, 2014; Kumari et al., 2022). According to (Patel et al., 2018), an increase in proline content was observed under abiotic-induced stress in *Triticum durum* treated with *Kappaphycus alvarezii* sap (K-sap). Overall, the accumulation of proline drastically improves the salt-stress tolerance of plants through different mechanisms, a part of being an osmoprotectant, proline can act as a regulator of redox status and attenuates cell acidity (Talaat and Shawky, 2014). Generally, proline level depends on the crop varieties, duration, and severity of salt stress. However, the accumulation of proline is not a specific indicator of salt stress, as it can be accumulated under different types of stress (drought, temperature) (Szepesi and Szollosi, 2018).

Phenolic compounds are a wide range of phytochemicals classified as secondary metabolites, endowed with diverse biological activities, including antioxidant (Sabraoui et al., 2020), antiproliferative (Paludo et al., 2022), hepatoprotective (Jia et al., 2022), and antidiabetic properties (Tatipamula and Kukavica, 2021). Phenolic acids are produced naturally in fruits and vegetable plants under various forms of stress (oxidative stress, salt stress, and drought stress) but also found abandonly in the grain fraction of cereals and crops (Tiozon et al., 2022). In Quinoa, phenolic compounds are present in two forms (soluble free or soluble conjugated to polysaccharides), with the predominance of gallic acid and ferulic acids followed by flavonoids (Pereira et al., 2020). Thus, free phenolic compounds contribute effectively to the sweet flavor of quinoa compared to the conjugated (Suárez-Estrella et al., 2018). In the current study, the TPC in quinoa seed ranged from 53.55 ± 1.23 to 841.01 ± 3.16 mg GAE/100 g of DW. In similar studies, (Han et al., 2019) studied the phenolic content of seven different varieties of coloured quinoa, the TPC content varied from 167.2 to 303.8 mg GAE/100 g of DW. Another work by (Mhada et al., 2020) reported values of TPC of 31.67 ± 7.26 and 105.85 ± 5.21 mg GAE/100 g of DW respectively in raw quinoa of two variety (Puno and Titicaca). In addition, at higher concentrations of salinity, TPC tends to increase significantly which is inconsistent with previous reports in which salinity enhances the biosynthesis of phenolic compounds (Petridis et al., 2012; Bistgani et al., 2019). Moreover, the finding of this study revealed that the application of amendments lowered significantly the TPC compared to the untreated quinoa. In a similar study, (Chehab et al., 2020) indicated that compost negatively influenced the phenolic content in olive fruits grown under saline conditions. Generally, environmental stress and particularly salinity stress regulates the biosynthesis of secondary metabolites. Organic amendments are a rich source of natural antioxidants such as vitamins, and amino acids which could attenuate salt-induced oxidative stress by modulating the antioxidant defense and the chelating activity of this crop (Liu et al., 2021).

Saponins are classified as anti-nutritional secondary metabolites of the glycoside family, naturally produced by plants as a response to a large spectrum of environmental abiotic stress;(Szakiel et al., 2011; El Aziz et al., 2019). Based on their chemical, saponins are divided into two main groups: steroidal and triterpenoid, their content varies from one plant to another depending on the variety, geographic location, and genetics (Cheok et al., 2014; Liao et al., 2017). Quinoa is a pseudo-cereal characterized by an abundant level of saponins, 86% is located mainly in the pericarp of the seed (Ando et al., 2002) their concentration in the upper layer is responsible for the bitterness or sweetness of the seed (Suárez-Estrella et al., 2018). In the current study, saponins’ level content raged from 0.22 ± 0.01 to 2.21 ± 0.07% of the dry weight at different salinity levels. (Mhada et al., 2020), reported similar values: 1.41 ± 0.05 to 2.09 ± 0.29% of dry weight evaluating the saponins content of raw quinoa (Puno and Titicaca). (El Hazzam et al., 2020) indicated a higher value of 6.34%. The bitter flavor of quinoa is a limiting factor and an organoleptic default to its introduction to the human diet. Therefore, different removal techniques have been reported in the literature: wet, dry, and genetics. To the best of our knowledge, this study is the first of its kind to describe the saponin response to organic amendment. When applied, organic amendments effectively reduced the saponin content at different salinity levels, such effect can be attributed to different mechanisms of amendments on attenuating the salinity-induced stress (Kumari et al., 2022). A study (Alam et al., 2020) suggested that organic amendments enhanced antioxidant activity and pea biosynthesis in pea crops grown under arsenic-contaminated soil. (Diacono and Montemurro, 2015) also indicated that organic amendments influence the physicochemical properties of soil due to the flocculation of minerals to organic polymers.

The damaging effects of salinity on plants, such as oxidative stress leading to harm to plant proteins, lipid peroxidation, and DNA damage, have been widely recognized. This occurs when excessive accumulation of reactive oxygen species (ROS) interferes with regular metabolism, (Petridis et al., 2012; Bistgani et al., 2019). The study employed three assays (DPPH, ABTS, and FRAP) to evaluate the antioxidant capacity of plant extracts, which involved the transfer of electrons from antioxidants to radicals to stabilize them. The findings indicated that quinoa seed exhibited notable antioxidant activity across various groups, regardless of the salinity level, while amendments significantly boosted the AOX compared to the untreated group. As previous studies have established, the production of phenolic compounds, which are secondary metabolites generated in response to abiotic stress, played a noteworthy role in the antioxidant capacity of a variety of plant species (Tang et al., 2015; Rusli et al., 2022). However, in our study, we did not observe any correlation between the total phenol levels in quinoa extracts and the antioxidant activity. The phenol content in these extracts showed a higher correlation with the results of the reducing power (FRAP) assay than with the DPPH and ABTS scavenging activity (**Table 7**). It has been noted by other researchers that the total phenol levels in extracts from pseudocereal seeds may not always be linked to their antioxidant activity. Hence, polyphenols from quinoa may be effectively reducing agents for metal ions but may not scavenge DPPH and ABTS efficiently due to steric hindrance (Yawadio Nsimba et al., 2008; Dini et al., 2010; Starzyńska-Janiszewska et al., 2016). The limited correlation observed between the overall phenolic content and the antioxidative activity implies that the primary antioxidants present in the examined seeds could be of a non-phenolic nature such as ascorbic acid, tocopherols, and carotenoids. This indicates that the understanding of the sources and mechanisms of antioxidant activity in quinoa is complex and cannot rely solely on total phenol levels. Further investigation is needed to identify the specific compounds responsible for the observed antioxidant activity in quinoa extracts. This could involve the isolation, identification, and quantification of individual polyphenols in quinoa extracts as well as more detailed analyses of their mechanisms of action.

## 5 Conclusions

This field experiment examined the ability of quinoa (*Chenopodium quinoa* Willd.) ICBA-Q5 variety to grow under different irrigation water salinity in combination with organic amendments. The finding of this study revealed considerable potential for improving quinoa yield under high salinity conditions through the application of organic amendments. Application of amendments alleviated salinity stress at the physiological level and ensured nutrient availability for plants. Further, saponin accumulation significantly decreased in response to the application of amendments, which represents an excellent opportunity to facilitate post-harvest operation and improve the grain quality of quinoa. Considering the several criteria such as yield, biochemical, and physiological, application of organic amendment is therefore suggested to improve quinoa productivity undersalt affected irrigated drylands such as South of Morocco.

## Data availability statement

The raw data supporting the conclusions of this article will be made available by the authors, without undue reservation.

## Author contributions

Conceptualization and methodology: AH, KD, AM, Talal Sabraoui. Data curation: AM, TS, AH. Investigation: AM, TS, IM, MB, FS, RE, MK, JZ. Chemical analysis: TS, AM, MI, KL, IM. Supervision: AH. Writing original draft: AM, TS, SR, AH. Review-editing: KD, CG AN. Resources mobilization: AH, CG, LK. All authors contributed to the article and approved the submitted version.
